# The Effects of Hypertension Treatment on Heart Rate Variability: A Scoping Review

**DOI:** 10.7759/cureus.91659

**Published:** 2025-09-05

**Authors:** Dylan Sehgal, Leonardo Bonifanti, Justin Tran, Matthew Lound, Anthony Manfredi, Harvey N Mayrovitz

**Affiliations:** 1 Medicine, Nova Southeastern University Dr. Kiran C. Patel College of Osteopathic Medicine, Fort Lauderdale, USA; 2 Osteopathic Medicine, Nova Southeastern University Dr. Kiran C. Patel College of Osteopathic Medicine, Fort Lauderdale, USA; 3 Osteoapthic Medicine, Nova Southeastern University Dr. Kiran C. Patel College of Osteopathic Medicine, Fort Lauderdale, USA; 4 Medical Education, Nova Southeastern University Dr. Kiran C. Patel College of Allopathic Medicine, Davie, USA

**Keywords:** antihypertensives, autonomic control, blood pressure, heart rate variability, hrv, hypertension, hypertensive medication, parasympathetic activity, sympathetic activity, vagus

## Abstract

This scoping review systematically gathered and synthesized evidence on the effects of antihypertensives on heart rate variability (HRV) in patients with hypertension (HTN). Antihypertensives are the cornerstone of blood pressure (BP) control, yet their influence on HRV, a marker of autonomic function and a predictor of cardiovascular outcomes, remains incompletely understood. A scoping review approach was selected to map the breadth of available evidence and identify knowledge gaps, focusing on randomized controlled trials to maximize internal validity. A scoping review was conducted in accordance with the Preferred Reporting Items for Systematic Reviews and Meta-Analyses extension for scoping review (PRISMA-ScR) guidelines. EMBASE, Ovid Medline, Web of Science, and CINAHL were searched for randomized controlled trials published between 1995 and 2021. Only English-language, peer-reviewed studies were included; gray literature was excluded.

Twenty-nine trials involving 2,230 participants were included, though designs were heterogeneous in duration and population characteristics. HRV was variably assessed using time-domain, frequency-domain, and nonlinear measures. Beta-blockers and non-dihydropyridine calcium channel blockers consistently enhanced parasympathetic activity and improved HRV. Angiotensin receptor blockers improved HRV in patients with concomitant metabolic syndrome, whereas alpha-1 antagonists showed limited but positive effects. Angiotensin-converting enzyme inhibitors and thiazide diuretics demonstrated minimal impact on HRV. No formal risk of bias assessment was performed, consistent with scoping methodology. The effect of antihypertensive therapy on HRV is drug-class dependent, with beta-blockers and non-dihydropyridine calcium channel blockers showing the most consistent benefits. Evidence-related gaps remain, particularly regarding under-studied drug classes, heterogeneous HRV measurement methods, and limited trial size. These findings highlight the potential role of HRV as a therapeutic consideration in HTN management and underscore the need for targeted research on under-studied drug classes.

## Introduction and background

In the United States, hypertension (HTN) is defined as an arterial blood pressure (BP) with a systolic value of 130 mmHg or a diastolic value of 80 mmHg or greater [1]. The U.S. definition was adopted in this review as it reflects the clinical practice context in which most of the included studies were conducted and aligns with the majority of current randomized controlled trials. While WHO and other international guidelines use different thresholds, the U.S. criteria provide a consistent operational definition for this evidence base. In the United States, approximately half of all adults have HTN, with only about a fourth having it under control [2]. Roughly 1.3 billion adults worldwide have HTN, with a likely rise to be seen in the future as the population ages [3,4].

Management of HTN varies drastically, whether it be modifying a patient’s lifestyle or administering a variety of different pharmacological therapies. The effectiveness of these therapies in controlling BP is patient-dependent and may influence heart rate variability (HRV) [5]. Lifestyle interventions are, for some patients, an effective way to lower BP and thereby reduce cardiovascular risk [6]. However, pharmacological treatment for HTN, concurrently with lifestyle modifications, has been shown to reduce BP for those unable to do it with lifestyle modifications alone [7].

HRV is defined as the variability in the time between two heartbeats. It is a commonly relied-upon marker to determine pathological conditions related to the cardiovascular system and gives insights into the interplay between sympathetic and parasympathetic effects [8]. It is not only a prognostic marker of cardiovascular health but also provides unique insights into the autonomic regulation in hypertension management, where sympathetic overactivity and parasympathetic withdrawal are common pathophysiological features. The autonomic nervous system (ANS) acts on the heart sympathetically through the cervicothoracic ganglion and parasympathetically through the vagus nerve [9]. HRV is often examined in the context of disease diagnosis. Low HRV has been reported to be associated with pathological conditions, including myocardial infarctions (MI), congestive heart failure (CHF), and HTN [10,11]. Importantly, changes in HRV of even modest magnitude (10-20 ms in RMSDD or SDNN) have been linked to meaningful differences in cardiovascular outcomes, making HRV a clinically relevant marker in hypertensive patients.

The links between changes in HRV and various morbidities are not fully understood. One report suggests that autonomic dysregulation, specifically sympathetic dominance or a reduction of parasympathetic activity, may be involved [12]. ANS modulation of the heart can be measured through several techniques, including ECG processing of EKG data. The simplest measure of HRV is based on time-domain analysis in which the time between successive R-R intervals of the EKG is determined. Common parameters include the standard deviation of R-R intervals (SDNN) and the root mean square of successive R-R intervals (RMSSD), with RMSSD primarily reflecting parasympathetic activity [13]. Frequency-domain methods analyze the spectral power of R-R intervals in ECG data, dividing it into bands that represent different aspects of autonomic control. The high-frequency (HF) band (0.15-0.40 Hz) predominantly represents parasympathetic (vagal) modulation, primarily driven by respiratory patterns. In contrast, the low-frequency (LF) band (0.04-0.15 Hz) encompasses both sympathetic and parasympathetic components, including influences from the baroreflex [14]. The absolute LF and HF powers are sometimes normalized to total spectral power (TP) and expressed as LFnu and HFnu, respectively, which estimate the relative balance between sympathetic and parasympathetic activity [15,16].

The normalized parameters help account for variations in total power and estimate the balance between sympathetic and parasympathetic activity [17]. LFnu and HFnu always sum to 100% and are closely related to the LF/HF ratio, though their exact physiological meaning is still debated [18]. While the ratio of LF to HF has been used to estimate the balance between sympathetic and parasympathetic activity, LF itself does not exclusively mirror sympathetic outflow [14,15]. HRV can be impacted by variable endogenous and exogenous factors, including lifestyle, physiological, pathological, environmental, age, gender, and non-modifiable factors [19,20]. Overall, these metrics enable clinicians and researchers to quantify autonomic function, assess the impact of antihypertensive therapies, and better understand the physiological mechanisms underlying cardiovascular risk. For example, beta-blockers and non-dihydropyridine calcium channel blockers are expected to augment parasympathetic activity, whereas agents such as thiazide diuretics are not thought to have strong direct autonomic effects. This biological rationale underpins the focus of the present review.

Currently, there is a lack of systematic reviews discussing the effects antihypertensives have on HRV. Studies exist defining specific classes of drugs used to treat HTN that examine the observed impact on HRV. A preliminary investigation of MEDLINE, the Cochrane Database of Systematic Reviews, and JBI Evidence Synthesis revealed no current or ongoing systematic reviews or scoping reviews on the topic. We specifically chose a scoping review methodology to map the scope of available randomized controlled trials' evidence, identify knowledge gaps, and clarify heterogeneity in study groups and populations, rather than restrict to a narrower set of studies suitable for meta-analysis. The present study aims to identify and summarize evidence on the effects of hypertensive therapies on HRV, highlighting patterns across drug classes to inform the understanding of autonomic modulation in HTN. This review will address the existing gap in the literature and aid in understanding the autonomic effects of antihypertensives. It is hoped that this will help guide clinician decision-making when tasked with providing the appropriate medical care.

## Review

Methods

Study Design and Search Strategy

A comprehensive search was conducted on September 15, 2024, across Embase, OVID Medline, Web of Science, and CINAHL following the Preferred Reporting Items for Systematic Reviews and Meta-Analyses extension for scoping review (PRISMA-ScR) guidelines [21]. The PRISMA-ScR guidelines were employed to ensure a methodical and precise approach to evidence evaluation for this scoping review. The search strategy was developed using keywords and index terms related to hypertension treatment and heart rate variability. A preliminary search of MEDLINE, the Cochrane Database of Systematic Reviews, PROSPERO, and JBI Evidence Synthesis was conducted, and no current or underway systematic reviews or scoping reviews on the topic were identified. The search was not updated before manuscript submission.

Following the JBI methodology for scoping reviews, we applied the PCC (Population, Concept, and Context) framework to define our search parameters. The target population consisted of individuals diagnosed with and treated for HTN who had undergone HRV measurements before and after treatment. The concept focused on the effect of HTN treatment on HRV, with the context being worldwide clinical studies involving humans with recorded HRV test results taken before and after receiving treatment for HTN by a licensed healthcare professional.

Eligibility Criteria

Studies were included if they involved humans, reported HRV measurements before and after hypertension treatment, were published in English, and were clinical trials or randomized controlled trials. Quasi-experimental designs, including non-randomized controlled trials, before-and-after studies, interrupted time-series studies, case-control studies, and analytical cross-sectional studies, were also considered to ensure a comprehensive mapping of evidence. Review articles, editorials, case reports, and meta-analyses were excluded. Gray literature, dissertations, and conference abstracts were not included.

References retrieved from the databases were managed using Rayyan and EndNote. In a two-tiered screening process, five independent reviewers initially performed a blind review of all references. Discrepancies were resolved through discussion and consensus. Data extraction focused on HRV parameters and BP values, aligned with the study objective of assessing autonomic effects of antihypertensive therapies; other autonomic markers or patient outcomes were not included. A standardized data extraction template was used, and discrepancies were resolved through discussion among reviewers. Although formal risk-of-bias assessments and inter-rater reliability statistics were not performed, study quality was considered descriptively during synthesis. The development of the search string using the four databases, along with the final numbers, is presented in Tables 1-4. Each table shows the search terms used in the process and the number of reports identified for each search string, and also the results when strings were combined. The last entry in each table is the final number of papers included in the preliminary screening process. Table 1 concerns the Ovid MEDLINE database. Table 2 pertains to the database Web of Science Core Collection. Table 3 is for the database Embase. Table 4 is for the database CINAHL.

**Table 1:** Search queries for Ovid MEDLINE database This table summarizes the exact search queries used for Ovid MEDLINE included in the study. For each row, the first column lists the search term number. The second column presents the full search query string as it was implemented in the respective database. The third column shows the number of articles retrieved after applying the expanded search terms and refinements, which included removal of duplicates and application of date or publication type limits as detailed in the Methods. This figure represents the final reduced set of articles per query

	Search term used	No. of records
1	Heart rate variability.mp	25,607
2	Heart rate variability.ab,kf,ti	25,607
3	Antihypertensive agents.ab,kf,ti	7246
4	Exp antihypertensive agents/	274,007
5	3 or 4	275,956
6	1 or 2	25,607
7	5 and 6	560
8	7 and “Randomized Controlled Trial”[Publication Type]	146

**Table 2:** Search queries for Web of Science Core Collection This table summarizes the exact search queries used for Web of Science Core Collection included in the study. For each row, the first column lists the search term number. The second column presents the full search query string as it was implemented in the respective database. The third column shows the number of articles retrieved after applying the expanded search terms and refinements, which included removal of duplicates and application of date or publication type limits as detailed in the Methods. This figure represents the final reduced set of articles per query

	Search term used	No. of records
1	TS=(Heart rate variability) AND TS=(antihypertensive agent*)	811
2	AND “Clinical Trials”	195

**Table 3:** Search queries for Embase database This table summarizes the exact search queries used for Embase included in the study. For each row, the first column lists the search term number. The second column presents the full search query string as it was implemented in the respective database. The third column shows the number of articles retrieved after applying the expanded search terms and refinements, which included removal of duplicates and application of date or publication type limits as detailed in the Methods. This figure represents the final reduced set of articles per query

	Search term used	No. of records
1	'Antihypertensive agent':ti,ab,kw	46,450
2	'Antihypertensive agent'/exp	3,757
3	#1 OR #2	1,055,859
4	'Heart rate variability':ti,ab,kw	36,423
5	'Heart rate variability'/exp	37,187
6	#4 OR #5	46,450
7	#3 AND #6	3,443
8	AND (‘clinical trial’/de OR ‘controlled clinical trial’/de OR ‘randomized controlled trial’/de)	654

**Table 4:** Search queries for CINAHL Database This table summarizes the exact search queries used for CINAHL included in the study. For each row, the first column lists the search term number. The second column presents the full search query string as it was implemented in the respective database. The third column shows the number of articles retrieved after applying the expanded search terms and refinements, which included removal of duplicates and application of date or publication type limits as detailed in the Methods. This figure represents the final reduced set of articles per query

	Search term used	No. of records
1	(Heart rate variability or HRV) AND antihypertensive agents	51
2	(Heart rate variability or HRV) AND (antihypertensive medications or blood pressure medication or antihypertensive agents or hypertension medication)	124
3	(Heart rate variability or HRV) AND (antihypertensive medications or blood pressure medication or antihypertensive agents or hypertension medication) expanded: related words and peer-reviewed	177

**Figure 1:**
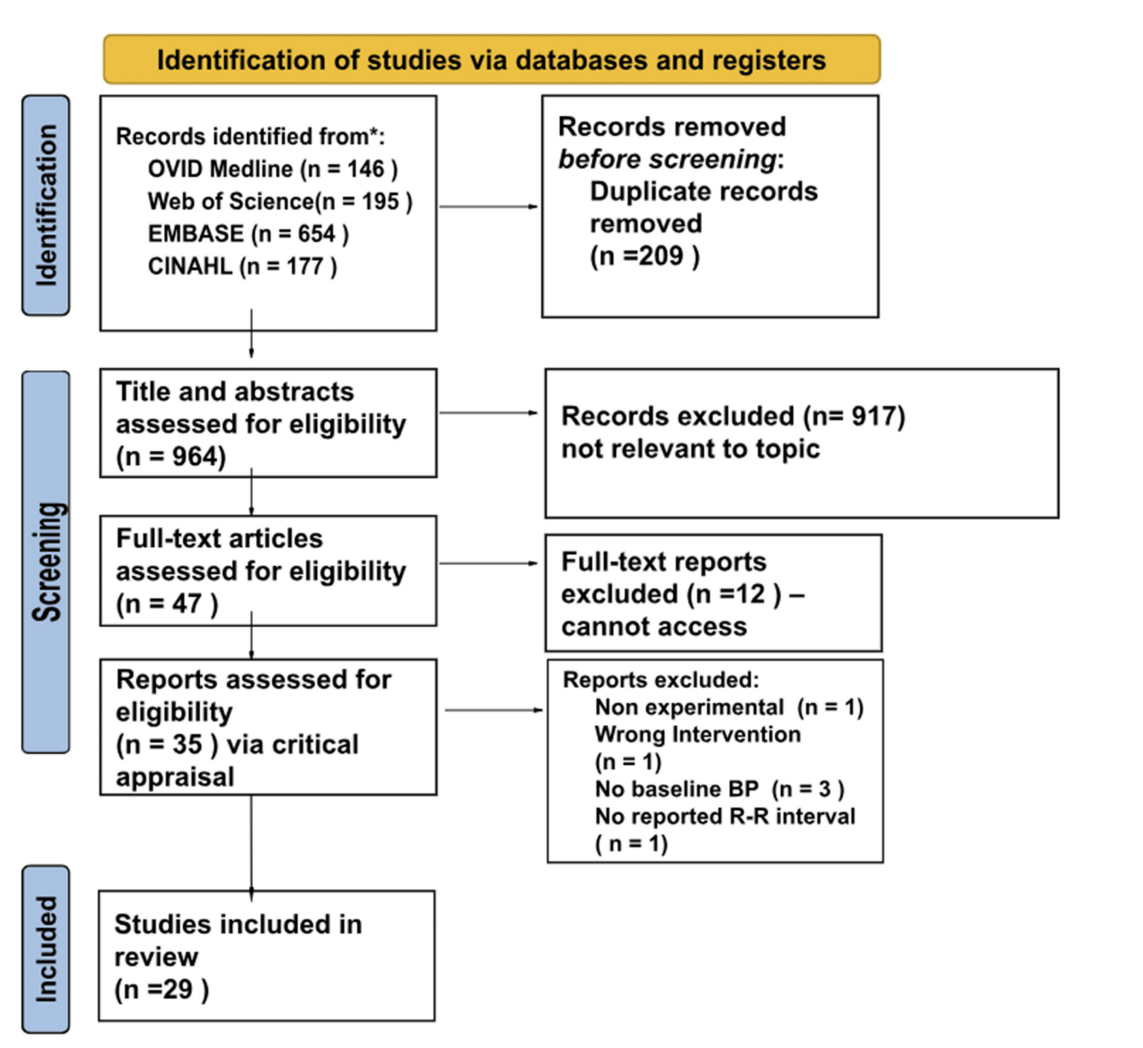
PRISMA-ScR flow chart depicting the study selection PRISMA-ScR: Preferred Reporting Items for Systematic Reviews and Meta-Analyses extension for scoping review

The PRISMA-ScR flow chart is shown in Figure 1.

Results

Of the 29 studies included, 20 reported significant effects on HRV as measured by spectral analysis or RMSSD. The following results are presented by antihypertensive class. 

Beta-Blockers

Two studies compared the effects of a beta-blocker vs. an angiotensin-converting enzyme (ACE) inhibitor on BP and HRV. In one, the comparison was between atenolol and cilazapril [22], and in the other, it was metoprolol vs. ramipril [23]. While in each study, both drug classes lowered BP to similar amounts, the beta-blockers had a more pronounced effect on HRV. Spectral analysis revealed that in patients treated with atenolol, the LF, LF/HF, and LFnu (normalized LF) were reduced, and HFnu (normalized HF) increased, suggesting a shift toward enhanced parasympathetic activity. Cilazapril did not cause changes in HRV measures related to sympathetic activity, as was indicated by unchanged spectral values. In the patients treated with metoprolol, the variability in R-R intervals increased, as did total HF spectral power, indicating improved autonomic balance. In contrast, ramipril did not have a significant effect on HRV spectral measures.

Other effects of beta-blockers on HRV parameters have been reported in a study that compared the effects of three antihypertensives [24]. In this study, the BP and HRV effects of fosinopril, an ACE inhibitor, irbesartan, an angiotensin receptor blocker (ARB), and atenolol were evaluated. While all three drugs significantly and equally decreased BP, only atenolol showed a significant increase in HRV, as measured by non-linear methods (SD1/SD2), reflecting short and long-term autonomic variability. Another study compared atenolol to the effects of a different ARB (losartan) [25]. In this study, atenolol treatment for six months resulted in a decrease in HRV as indicated by decreased total, LF, and HF spectral powers. Contrastingly, for patients treated with losartan, the same spectral measures indicated an increase in HRV. This differential response occurred despite both drugs resulting in similar BP reductions, highlighting that the direction of HRV change may vary by beta-blocker type, treatment duration, and baseline autonomic status.

A comparison between losartan and the beta-blocker bisoprolol also showed a statistically significant reduction in BP for both drugs. Despite the equivalency of BP reductions, the HRV responses were different. TP was reduced in patients treated with bisoprolol, whereas there was no HRV effect on patients treated with Losartan [26]. A reduction in TP was also found in patients treated with a combination of bisoprolol and hydrochlorothiazide [27]. However, in this study, the reduced TP was due mainly to a reduced LF component, whereas the HF component increased. The combined spectral component changes resulted in a reduction in the LF/HF ratio, suggesting a shift in autonomic balance toward enhanced parasympathetic (vagal) activity and diminished sympathetic modulation, which is generally considered beneficial in HTN. The authors attribute these changes to the reduction in HR associated with beta-blockade; however, the sustained decrease in LF power after adjusting for HR suggests a direct sympatholytic effect of beta-blockers. This autonomic profile suggests that beta-blocker-based regimens may suppress sympathetic activity more effectively than newer antihypertensive combinations, which could influence cardiovascular risk outcomes by altering autonomic regulation in a manner that may not provide optimal risk reduction compared to other agents [27].

The possible role of patient age on HRV effects was investigated using the beta-blocker Betaxolol as initial treatment of male patients with HTN-related tachycardia and comparing outcomes on younger vs. older patients [28]. While BP decreased significantly in both groups, the reduction was more pronounced in older patients. Furthermore, the younger group experienced a statistically significant increase in HRV, but the older patients did not. This HRV was measured using the SD1/SD2 ratio, which is a nonlinear HRV index that reflects the balance between short-term (SD1) and long-term (SD2) variability, and thus sympathetic and parasympathetic modulation. [28]. It should be noted that, within the studies included in the review, this was the only one that stratified outcomes by age.

A comparison of the effects of two beta-blockers (bisoprolol and atenolol) showed both to significantly reduce BP and also decrease the LF/HF ratio [29]. The LF/HF decrease may indicate reduced sympathetic nervous system activity or an increase in parasympathetic (vagal) modulation of HR. This shift in autonomic balance is considered beneficial, as excessive sympathetic activity is associated with adverse cardiovascular outcomes in HTN [29]. However, it is important to note that not all studies consistently linked LF/HF reductions to clinical outcomes, and the direction of HRV change varied across beta-blocker types and study conditions.

Not all studies demonstrated significant effects of beta-blockers on HRV. Two of the reviewed studies reported no significant changes in HRV parameters despite effective BP reduction with beta-blocker therapy [30,31]. Overall, these findings highlight the heterogeneity in HRV responses, measurement methods, and potential influences such as drug type, duration, baseline autonomic status, and adjustment for heart rate. Non-linear HRV measures were included in some studies, but not consistently assessed all beta-blocker trials.

Calcium Channel Blockers (CCBs) 

The effects of CCBs on HRV appear to depend on the type used. Several studies compared the effects of non-dihydropyridine CCBs (Verapamil and Mibefradil) to dihydropyridine CCBs (felodipine, amlodipine, and nifedipine) on BP and HRV [32-34]. All reported that each drug pair reduced BP and increased HRV, with non-dihydropyridine CCBs generally producing greater HRV increases.

In another study, the effects of the non-dihydropyridine CCB diltiazem on 24-hour BP, HR, and autonomic nerve activity were evaluated [35]. Results indicated that diltiazem significantly decreased BP, HR, and the LF/HF ratio. The decreased LF/HF was interpreted as diminished sympathetic nervous system activity, with improvements in HRV partially correlated with the reduction in HR. However, an opposite effect on parasympathetic effects was reported using the dihydropyridine CCB lacidipine, which showed a significant decrease in RMSSD, a time-domain marker of vagal activity, suggesting possible sympathetic predominance or parasympathetic withdrawal; differences may reflect drug-specific effects, dosage, or study population, although this was observed in a single study [36].

To study the possible effects of the drug release mechanism on HRV, the CCB nifedipine-CR and nifedipine retard were compared [37]. It was reported that nifedipine retard produced a significant reduction in 24-hour and daytime average values of LF and HF, while nifedipine CR affected only the nighttime LF component and did not change the HF component. These findings, reported in a single study, suggest that the retard formulation may exert a more pronounced modulation of both sympathetic and parasympathetic activity throughout the day, whereas the CR formulation has a more limited impact, particularly on parasympathetic (HF) tone [37]. Furthermore, another study demonstrated that amlodipine induced nighttime HF reduction but increased LF and the LF/HF ratio during the day, indicating a shift toward sympathetic dominance; however, the clinical significance of these LF/HF changes was not explicitly reported. In contrast, telmisartan enhanced parasympathetic tone during both day and night, and ramipril increased daytime HF and lowered the LF/HF ratio without elevating norepinephrine [38].

A comparison of HRV effects of diltiazem vs. beta-blockers combined with either ARBs or ACE inhibitors showed that the diltiazem-treated group demonstrated a significant improvement in HRV parameters, including SDNN and RMSSD based on 24-hour ECG monitoring [39]. This improvement was observed despite comparable BP reductions and appears partially related to HR reduction, suggesting both HR-dependent and HR-independent contributions. Those treated with diltiazem also had a greater HR reduction and superior gains in exercise tolerance, suggesting the enhanced HRV may contribute to improved functional capacity.

Despite the reports of improved HRV associated with CCBs, some studies reported no significant effects. In one study, there were no significant changes in HRV with a dihydropyridine CCB [40]. In another study, it was observed that amlodipine treatment led to a non-significant decrease in LF power and had no effect on the HF band [41]. Although these reductions in LF suggest a possible decrease in sympathetic activity, the lack of significant changes in overall spectral measures and baroreflex sensitivity limits definitive conclusions [41]. Another study conducted in patients with mild-moderate essential HTN discovered neither amlodipine nor fosinopril caused significant long-term alterations in HRV parameters, despite both drugs being effective in reducing BP [42].

Angiotensin Receptor Blockers (ARBs)

In contrast to CCBs, the effects of ARBs seem to vary and may depend on patient characteristics. While some studies indicate that ARBs can improve HRV parameters, especially in patients with baseline autonomic dysfunction, only a limited number of studies reported significant improvements. For example, in one study, HRV in patients with and without metabolic syndrome was assessed. It was reported that the ARB (valsartan) lowered BP in both groups, but only in patients with metabolic syndrome was there a significant increase in HRV, indicated by higher SDNN, RMSSD, and TP. These improvements were observed despite similar BP reductions in both groups, suggesting that HRV changes were not solely dependent on BP lowering. The beneficial effects were primarily seen in time-domain measures and TP, whereas frequency-domain changes were less consistent. This suggests that, regarding HRV, ARBs might be particularly beneficial in populations with greater autonomic impairment [43].

Alpha Blockers

Alpha-blockers demonstrated slightly positive effects on HRV. A single study evaluated how extended-release doxazosin impacts autonomic modulation in patients already on a multi-drug regimen for uncontrolled HTN [44]. They found a significant increase in resting spontaneous baroreflex sensitivity (BRS) and 24-hour short-term and total HRV. The study assessed HRV over the short-term treatment period, and long-term persistence of these effects was not reported. BRS is a noninvasive measure of autonomic function that reflects the heart’s ability to adjust its rate in response to spontaneous changes in BP and is considered a functional marker of parasympathetic activity and autonomic responsiveness. Furthermore, there was a significant association between the degree of change in diastolic BP and short-term HRV, indicating some correlation between BRS improvements and HRV measures [44].

Angiotensin-Converting Enzyme (ACE) Inhibitors

The effects of the ACE inhibitor enalapril on HRV and autonomic nervous system function appear to vary according to the baseline autonomic profile of the patient [45]. Enalapril significantly reduced BP, and in individuals with elevated sympathetic activity, it also reduced the LF spectral component of HRV. In contrast, patients with predominant vagal tone exhibited increased LF and decreased HF. This bidirectional modulation suggests that enalapril may contribute to restoring sympathovagal balance by either dampening excessive sympathetic drive or moderating parasympathetic predominance, thereby promoting autonomic normalization in hypertensive patients [45].

However, several other studies found no significant changes in HRV frequency domain markers with ACE inhibitor therapy despite drug-related reductions. These studies report results as increased LF components or decreased HF components, reflecting sympathetic and vagal tone, respectively, without clear evidence of bidirectional modulation. This suggests that the angiotensin system may play a limited role in autonomic control, as evidenced by the lack of spectral changes associated with ACE inhibitor treatment [46,47].

Thiazide Diuretics

Limited data were available regarding thiazide diuretics. In a single comparative study of Natrilix SR (thiazide diuretic), candesartan (ARB), and amlodipine, treatment with Natrilix SR and amlodipine was associated with decreased HRV, particularly at night. The reduction in HRV was significantly correlated with decreases in systolic BP variability at night (p=0.04) but was not significantly associated with reductions in mean BP after adjustment (p=0.06). The study did not report whether the HRV reduction was dose-related, and no studies evaluated combination therapy with thiazides and their effects on HRV. Overall, the decrease in HRV generally reflects impaired autonomic regulation rather than a beneficial effect on autonomic modulation [48]. Further studies are needed to confirm these findings.

Discussion

This scoping review examined the reported effects of various antihypertensives on HRV, an important marker of autonomic function and cardiovascular prognosis. Across the 29 included studies, most reported significant HRV changes with treatment, with 20 studies showing measurable effects. Findings varied markedly by drug class, patient characteristics, and study methodology. Approximately one-third of studies found no measurable effects, underscoring the complexity of pharmacologic management of autonomic tone in HTN.

Beta-Blockers: Consistent Autonomic Shifts but Not Uniformly Beneficial

Beta-blockers generally showed a positive influence on HRV. They demonstrated the most consistent association with improved HRV indices, particularly reductions in the LF/HF ratio and increases in HF power, as well as improvements in non-linear indices such as SD1/SD2 reflecting a shift toward parasympathetic predominance. These findings are in line with prior mechanistic studies showing beta-adrenergic blockade attenuates sympathetic drive. Effect sizes varied across studies, and younger patients appeared more responsive, suggesting age-related autonomic plasticity may influence treatment effects. Sample size variability, treatment duration, and concomitant medications likely contributed to heterogeneity.

However, some trials noted reductions in total power or mixed results depending on the comparator (e.g., losartan), highlighting that autonomic effects may not be uniformly favorable and can depend on the beta-blocker used, dosing, and patient age. Not all studies consistently link HRV changes to clinical outcomes, emphasizing that HRV remains a surrogate marker rather than a confirmed predictor of hard endpoints.

Calcium Channel Blockers: Varying Effects Based on Type

The influence of CCBs appeared to depend more on subclass than on BP reduction. Non-dihydropyridines generally enhanced parasympathetic tone more than dihydropyridines, with improvements in time-domain (RMSSD, SDNN) and frequency-domain indices (HF, LF/HF) where reported, a pattern supported by prior reports linking HR reduction to improved HRV. Drug formulation (controlled-release vs. retard) also altered autonomic outcomes, implying that pharmacokinetics may meaningfully shape autonomic effects. Magnitude of effect varied, follow-up periods differed across studies, and sample sizes were small, potentially influencing heterogeneity. Concomitant medications were inconsistently reported, which may have affected HRV outcomes. Nevertheless, inconsistent findings with some dihydropyridines, ranging from no change to possible sympathetic activation, indicate that this class exhibits heterogeneous autonomic effects.

*ARBs and ACE Inhibitors: *Selective and Context-Dependent Benefits

ARBs and ACE inhibitors generally produced smaller or less consistent HRV changes. Time-domain indices (SDNN, RMSSD) and TP were more frequently reported than frequency-domain measures, which showed greater variability. When improvements occurred, such as with valsartan in patients with metabolic syndrome, they often appeared in populations with baseline autonomic impairment, suggesting a role in restoring rather than enhancing autonomic function. These HRV improvements occurred independently of BP reduction, highlighting intrinsic autonomic modulation. Sample sizes were small, follow-up periods varied, and baseline therapies differed across studies, which may influence observed effects. The bidirectional HRV modulation observed with enalapril supports this context-dependent mechanism.

Alpha-Blockers and Thiazide Diuretics: Preliminary but Promising Evidence

Evidence for alpha-blockers was limited but encouraging, with doxazosin improving BRS and HRV measures in patients on multi-drug regimens. The effects were assessed over short-term follow-up, and long-term persistence remains unknown. The sample size was small, and these findings should be interpreted with caution. While promising, these results require replication before clinical relevance can be established.

Data on thiazide diuretics remain too scarce for definitive conclusions; preliminary findings suggest possible HRV reduction, with changes correlated with nighttime systolic BP variability but not with mean BP, and no dose-response or combination therapy data were available. This warrants further investigation given their widespread use in hypertension management. Overall, these findings contrast with the relatively consistent improvements in HRV observed with beta-blockers, highlighting the more modest or heterogeneous autonomic effects associated with alpha-blockers and thiazides.

Integrating Findings With Clinical Context

Taken together, these findings reinforce that HRV responses to antihypertensives are not solely determined by BP reduction. Drug class, subclass, formulation, and patient factors (age and metabolic status) emerge as key modifiers of autonomic outcomes. Sex differences were rarely reported, limiting conclusions regarding gender-specific effects. This suggests that in patients with concomitant autonomic dysfunction, class selection may be clinically relevant beyond BP control. However, the heterogeneity of existing studies limits the ability to draw prescriptive recommendations, and current HTN guidelines do not yet integrate HRV into therapeutic decision-making. While HRV is not yet incorporated into guidelines, its modulation could represent an important area for personalized therapy, particularly in patients with autonomic dysfunction.

Limitations

This scoping review is limited by the diversity of patient populations, HRV measurement techniques, and the relatively small number of studies with reportable data on HRV measurements and variations in classes of antihypertensives. Despite these constraints, the findings suggest that certain antihypertensives have a significant effect on HRV, although the magnitude and duration of these effects require further elucidation.

Implications for future research

Standardized HRV measurement protocols, longer-term follow-up, and stratified analyses by patient phenotype are needed to clarify whether targeting HRV improvement can translate into reduced cardiovascular risk. Particular attention should be given to time-domain and frequency-domain HRV measures, as well as nonlinear indices, to determine which domains are most informative. Patient subgroups such as individuals with diabetes, older adults, and those with resistant HTN may be prioritized in stratified analyses to better understand differential autonomic responses. Further studies should report quantitative effect sizes, control for confounders (baseline therapy, concomitant medications, lifestyle factors), and integrate mechanistic insights linking sympathetic and parasympathetic pathways to specific drug classes. Given the emerging role of autonomic dysfunction in predicting adverse outcomes, integrating HRV endpoints into HTN trials could help bridge the gap between physiological modulation and clinical benefit, although HRV remains a surrogate marker, and direct links to hard cardiovascular outcomes such as MI, stroke, or mortality remain to be fully established.

## Conclusions

This scoping review examined the effects of antihypertensives on HRV before and after treatment in patients with HTN. Beta-blockers and non-dihydropyridine CCBs generally demonstrated improvement in HRV indices such as SDNN, RMSSD, and LF/HF ratio, enhancing parasympathetic activity. Other drug classes showed minimal or inconsistent effects, with heterogeneity across studies and some benefits in select populations. It should be noted that assessment of autonomic function is not currently incorporated into HTN guidelines, and while these findings are promising, clinical implementation will require further validation. These findings emphasize the importance of considering both BP control and autonomic function in therapy decisions. Further studies should aim to elucidate drug-specific mechanisms affecting HRV, evaluate standardized measurement protocols, long-term outcomes, and determine whether HRV-guided therapy can enhance risk stratification and personalize antihypertensive treatment.
